# Summering

**Published:** 1879-06

**Authors:** 


### SUMMERING.

If you want the luxury of rest. and that is what our citizens most need, go to a farm-house, several miles from a railroad station, or steamboat landing, which promises nothing more than cleanliness and plain fare, whose occupants make no pretensions to have descended from the Great Mogul; or to have been once rich, and lost all by going security; who don't own to having any rich relations, nor to have had as boarders, formerly, the Queen of Sheba, or Cleopatra, or Victoria Regina. If you happen at a farm-house where they begin on "this line," you may be sure that will be their only "line," and their biggist line, to the end of the chapter, and had better "move" right off, bag and baggage, even if it is dark, and camp out that night in the corner of a fence, a mile off. Why do our people submit so passively to the robberies and impositions of Long Branch, Saratoga and Cape May? What of rest, enjoyable rest, do any of such places give to a wearied body, to an over-worked brain, and a constitution ready to break by the excessive toils of city life. Go, reader, where you can dress in your commonest clothing; and you poor over-worked wife don't take that everlasting "sewing" along with you; resolve not to take a single stitch during your entire absence; let your only "sewing apparatus" consist of a ball of yarn, a skein of silk, and two needles; leave your thimble at home; if you sew on all the buttons, and keep the stockings darned, that will be quite enough. Sleep from dark until sunrise; be out and about on foot or horse, in carriage, rowing, boating, swimming, botanizing; or with hammer and microscope study the rocks and read the histories of the localities from ages before the Flood, as you can do; climb mountains, explore caves; in short, do anything and everything which will entertain, instruct, or gratify yourself, or others, from breakfast until noon, and from dinner to sun-down; so that when you come home to dinner, you will be as hungry as a bear, and bacon, cabbage and corn-bread will taste sweet to you; after dinner you go again, that when you come home at sun-down, you will sigh for the bed, hardly taking time to have a dry cracker and a cup of tea, all that you ought to have, and all that night you will sleep like a baby, and next morning you will feel hungry enough to eat your wife and children, and so happy and good natured that life would have new charms for you. Rest from care, from business anxiety, from dress, and the needle, from the conventionalities of society, these are what city people need during summer, and they most certainly are not to be had at the seaside or the Spa.

A grand good idea for all concerned, would be for the managers of the Pacific Railroad to take persons for half fare to the utmost limit of their line, beyond even Cheyenne; this would make the road talked about, would bring it to the attention of the public.

Dr. Kirkbride, of the Pennsylvania Hospital for the Insane, in his report to the Legislature, says, one seventh of the male patients had no regular occupation at the time of attack, while for 1850 the most numerous class were those who had been in some way engaged in agriculture, either as farmers, farmers' wives or daughters. This proportion is not invariable, for in 1862 the same gentleman reports, that of 3947 insane, there were 297 farmers, 170 of their wives, and 95 of their daughters —about one seventh of the whole were from the farm! Of the 4014 patients in the Central Lunatic Asylum of Ohio, as reported to the Legislature for 1862, 1108 were from the farm! The statistics of the insane in Massachusetts show that the largest number of cases were of farmers' wives.

Nor do farmers live the longest. Travelers and natural philosophers average a greater age. The clergyman who devotes his life to study and late hours, who spends three fourths of his existence in-doors, who does not average two hours' daily exercise in twenty-four, who is compelled to an inactivity of body which would seem enough to undermine any constitution, to say nothing of the many depressing influences connected with his office, in listening to the troubled, in counseling the sick, and in waiting upon the dying and the dead, even he often survives the farmer, who rises with the lark to breathe the pure out-door air, whose undisturbed nights, whose supposed independence of the world, whose table is thought to be spread every day with the freshest butter from the dairy and the new-laid eggs, with pure, rich milk from the spring-house, all cool and sweet, vegetables just dug from the ground or pulled from the vine, and melons taken from the garden, berries from the bending bushes, and fruits, luscious, perfect, and ripe, from the orchard within the hour; in short, a class of persons whose whole surroundings are universally believed to be the synonyms of quiet, plenty, and independence, and which would seem to be a full guarantee of a healthful and happy old age; do not attain it as often as some other classes whose habits and modes of life are not, other things being equal, as favorable to longevity.

In an official report made before the Ohio Legislature, in February, 1863, it was stated that the unmarried exceeded the married by seven hundred and sixty. A sufficient reason for this result is found in the fact, that the mental and moral faculties are called into constant activities in married life, while the very multiplicity of its cares, and their changing nature, so diversify the mental operations, that the brain can not dwell long on one subject The report very significantly adds: "Those whose lives are devoted to physical labor appear to be far more liable to insanity than those whose occupation calls for a larger exertion of the mental faculties."

The want of health, and sanity, and longevity among farmers is not a necessity. Two thousand years ago, when life was less artificial, the oldest, and wisest, and best of men were farmers; their names have come down to us through the long ages intervening, with honor, and they are held in respect in all civilized lands. An able writer in the *Atlantic Monthly* for April, 1863, says: "Xenophon (the most renowned of the world's generals) gained a strength in his Elian fields that carried him into the nineties; Cato lived to be over eighty; Varro wrote his book out by Tusculum at eighty, and survived to counsel with Fundania ten years more Pliny, too, the Elder, who, if not a farmer, had his country-seats, and left very much to establish our acquaintance with the Roman rural life, was a hale, enduring man, of such soldierly habits and large abstemiousness, as to harvest a good four-score, if he had not fallen under that murderous cloud of ashes from Mount Vesuvius in the year seventy-nine." And who has not read of the good, and great, and wise old Cincinnatus of Roman story?

These examples, with thousands of others in our own day, confirmed by the observation of every intelligent man, prove that a sane mind in a sound body naturally belongs to a farmer's life; and when it is not so, it is because of adventitious circumstances — to wit, a want of intelligence, a want of mental activity on a variety of subjects, and a want also of that intelligent attention to the physical nature, to those laws of health as pertaining to domestic life in relation to personal and habitational cleanliness, to dress, to habits of rest, and sleep, and work, to eating and drinking, and, not least, to a rational cookery, all of which are essential to high health under any circumstances, but more especially so in connection with that artificial mode of life which has so unfortunately extended itself from the city to the town, and village, and hamlet, and to the log-cabin itself.

Not one farmer's wife in fifty is a good bread-baker, and yet bread is the staff of life. The unwise and wicked penuriousness of multitudes of farmers, sends the best and freshest and most luscious articles for the table to market, while he feeds himself, wife, children, and hired help on the milk and butter returned from town, or on the defective fruits and melons of the orchard and the field and garden. There are tens of thousands of them who sit down to their tables for weeks at a time without having a single dish of fresh meat to break the odious sameness of pork and beans, of cabbage and bacon, the hams being sent to market, and spring chickens, and splendid turkeys, and fat young ducks, swarm in the yard, and around the barn, and scatter over the fields—in fact, are to be found everywhere except on their own tables. This is a shame and a disgrace and a sin; because good food, well prepared, is second in importance to nothing that can be named about a farm-house. It is perfectly certain that whatever may be the discomforts, and annoyances, and miseries found in farmers' families, be they more or less, be they physical, mental, moral, social, or domestic, a very large share of them come from bad food, illy prepared, and irrationally eaten.

Let all, especially the young, who look to farming as the future pursuit of life, and who desire to avoid a large share of the ordinary discomforts, privations, unhappiness, and want of health which too often befall so worthy and so large a class of society as farmers' families are, remember these two cardinal suggestions:

First. Never purchase more land for farming purposes than can be paid for without borrowing.

Second. Never attempt to cultivate more than can be thoroughly done with the help which can be readily commanded; for one acre will yield more with a given amount of well-expended labor than two acres will yield with the same.

### HARDSHIPS OF FARMERS' WIVES.

A sad record is it, and short! but its details would fill whole shelves of a library, more intensely interesting than any tale fancy ever told, as found in an official report for 1862, made to the Legislature of an agricultural State, that of six hundred and seven patients in an insane asylum, thirty-nine were farmers' wives, sixteen farmers' daughters; no other class of wives or daughters was half as numerous! and this in spite of all that has been said and sung of the dairy-maid, so ruddy of cheek, where the roses and the lily vie, so lithe of limb, whose breath, as pure as the air of the morning, whose laugh, as merry as the voices of the birds in the wood, and whose step as springy and elastic as the new-made bow; all these bright fancies vanish like mists of the morning before a summer's sun, in face of the hard, dry, statistical line written above; and there comes up another vision, not of youth and beauty and innocence and exuberant health, but that of the pale and wan and haggard face, half covered with long black hair, and coal-black eyes peering hotly on you from behind the bars and grates of a dark prison-house!. True, happily true is it, that this state of things is "not to the manor born;" is not part and parcel, necessarily, of the farm-house; it is not an inherent calamity; it is only an accidental circumstance, which can be remedied promptly and forever! and it is because of this delightful truth these pages are written.

The remedy for the startling evil which the official statement made in the beginning brings to light is in the husband and mother. Let the farmer feel that his wife is an equal partner on the farm, and as such is entitled to as high consideration as he claims to himself. He should have a jealous care for the "good name of the house." Let him feel that what degrades his wife degrades himself, that whatever weakens her authority weakens his own power of success; and that in the great struggle of life they must of necessity rise or fall together. But while he cherishes these views as a business matter, as a practical thing of profit and loss, let him make an effort in another direction, not considering his wife merely as his partner in business, but as the love of his youth, who having, in a perfect abandon of trustingness, thrown herself in his strong arms to guide and protect and sustain through life's long journey, has claims on him for these, stronger than any tie other than that which binds man to Divinity; so strong, indeed, that inspiration has declared it, that in heaven was it made, and by heaven only can it be unloosed; and feeling thus, let him see in the wife of his bosom, though she may be all wrinkled with age, only the fair and loving and the fondly trusting girl as she appeared at the moment of saying "I will" many years agone. Let the mother also busy herself in teaching her daughters what they ought to do, what they have a right to expect in the marriage relation; and above this even, let her inculcate on her sons, day by day, with wisdom and tenderness, charging them lovingly to remember when she is dead and gone, that by all the respect and reverence and affection which they may have for her memory, to treat for her sake the wife of their bosom with all that affection and tenderness and consideration and sympathy which they would have their father show her if she could but be brought back again, or which they themselves would gladly show her if they had the opportunity.

Many a farmer's wife is literally worked to death in an inadvertent manner; from want of reflection or consideration on the part of her husband. None can understand better than he, in plowing or sowing, or harvest-time, that if a horse gets sick, or runs away, or is stolen, another must be procured that very day, or the work will inevitably go behind-hand. He does not carry the same practical sense into the kitchen when the hired help leaves without warning, or becomes disabled; although he knows as well as any man can know, that "the hands" will expect their meals with the same regularity, with the same promptness, and with the same proper mode of preparation; but instead of procuring other "help" on the instant, he allows himself to be persuaded, if the "help" is sick, she will get well in a day or two, or week at farthest, and that it is hardly worth while to get another for so short a time; if the "help" has taken "French leave," his mind fixes on the fact that it is a very busy time, and neither he nor a single hand can be spared; or that, in the course of a week, some one will have to go to town for some other purpose, and both these matters can be attended to at the same time; meanwhile, the wife is expected not only to attend to her ordinary duties as usual, but somehow or other to spare time to do all that the cook or washerwoman was accustomed to; that is, to do the full work of two persons, each one of whom had already quite as much labor to perform as she could possibly attend to. The wife attempts it; by herculean efforts all goes on well; the farmer perceives no jar, no hitch, in the working of the machinery; and because no complaint is uttered, thinks that every thing is going on without an effort; meanwhile time passes—and infinite shame on some of them!—they begin to calculate how much has been saved from servants' wages, and how much less food has been eaten; and because still no complaint is made, the resolution quietly forms in the mind to do nothing until she does complain; but, before that takes place, she falls a victim to her over-exertions, in having laid the foundation for weeks and months of illness, if not of a premature decline and death. Sincerely it is believed that these statements ought to be written in large letters above the mantles of half the farmers of the country; and if over the other half also, it would not be labor lost in favor of many a heroic and uncomplaining, but outraged farmer's wife and daughter.

The plain fact is, no farmer who is even half a man, will allow himself to have any help on the farm until he has first supplied a "help" for his wife in the kitchen; for when any man sets out to make a living by farming, there is always quite enough for two women to do,—even if there are no children, nor any prospect of any. No one woman can possibly do all the work of a farm which belongs to her department, even if the only "hand" is her husband; for there is cooking and washing, and sewing and mending, and beds to make; rooms to sweep and floors to wash, and cows to be milked, and butter to be made, and the small gardening to be attended to, and provision to be made for the company that will occasionally call in, and that ought to call in, to break the tedium of the livelong day, the husband in the fields, and not a living soul to exchange a human word to! Whatever young farmer thinks that one woman could or ought to attend to all these matters, even on a one-hand farm, is utterly destitute of all chivalry, and disgraces his own manhood, in that it looks strongly to the fact that he considers his wife more a kind of beast of burden than a rational, social, and intellectual being.

The generous-hearted and manly farmer will steadily bear in mind the great practical fact, that only half his duty has been performed toward the wife of his bosom when he has removed the physical hardships of her lot as far as his means will allow, for these bear chiefly on her physical nature in the direction of shortening her life by but a few years only; the greater evil of a living death behind the bars of a mad-house is the result.

